# Oxidative stress due to *Mycobacterium avium* subspecies *paratuberculosis* (MAP) infection upregulates selenium-dependent GPx activity

**DOI:** 10.1186/s13099-016-0090-8

**Published:** 2016-03-02

**Authors:** Ahmad Qasem, Ahmad Abdel-Aty, Huda Abu-Suwa, Saleh A. Naser

**Affiliations:** Burnett School of Biomedical Sciences, College of Medicine, University of Central Florida, 4110 Libra Drive, Orlando, FL USA

**Keywords:** Crohn’s disease, *Mycobacterium paratuberculosis*, MAP, Glutathione peroxidase, Oxidative stress, Type I diabetes

## Abstract

**Objective:**

This study was designed to determine the relationship between *Mycobacterium avium* subspecies *paratuberculosis* (MAP) infection and selenium-dependent glutathione peroxidase (GPx) activity, in the blood of humans and cattle infected with MAP.

**Design:**

MAP infection status and GPx activity were determined in sera from 42 cattle, a group of 27 patients with Crohn’s disease and 27 of their healthy biological relatives, and a group of 66 subjects with various diseases other than Crohn’s disease and 34 non-related healthy subjects.

**Results:**

GPx activity was significantly higher overall in the case of MAP infection in both humans and cattle. The mean value for GPx activity was 1.59 ± 0.65 units/ml in MAP positive cattle compared to 0.46907 ± 0.28 units/ml in healthy cattle sera, where a unit was defined as one mmol/minute (P < 0.01). The mean value of the GPx activity in MAP negative humans clinical sera was 0.42367 ± 0.229 units/ml compared to 0.80941 ± 0.521 in MAP positive sera in a study comparing Crohn’s disease patients to their healthy relatives. The mean activity in MAP negative humans was 0.4702 ± 0.1299 compared to 0.6510 ± 00.1665 units/ml in positive samples in a randomized field study of 100 subjects.

**Conclusion:**

This study demonstrated a strong correlation between MAP and the elevation of GPx activity. This was especially evident in Crohn’s patients, which further supports the association of MAP and Crohn’s disease. GPx activity may also be used to predict MAP infection status and to show that Crohn’s disease patients who are infected with MAP have higher tendency to develop oxidative stress than Crohn’s disease patients who are negative for the bacteria.

## Background

*Mycobacterium avium* subspecies *paratuberculosis* (MAP) is implicated in the etiology of multiple diseases including Crohn’s disease (CD) and both type I and type II diabetes mellitus (DM) in humans [1–4]. Several studies have proposed that MAP is a causative factor in type 1 diabetes due to cross-reactivity between MAP and human proteins [5, 6]. It is also known to be a causative agent of Johne’s disease, a bovine disease similar to CD in humans [7]. MAP is an obligate intracellular pathogen, living inside the macrophages of the infected host [8]. MAP increases the suitability of the macrophage as a host and prevents its own destruction by preventing the acidification of the phagosome [9, 10]. This is done by preventing the fusion of the lysosome and the phagosome into the phagolysosomal complex [9, 10]. MAP is also resistant to destruction even in an acidified, mature phagolysosome [11]. The primary mechanism for the destruction of MAP resistant to phagolysosomal degradation is the induction of apoptosis of the infected macrophage through a tumor necrosis factor α (TNF-α) dependent mechanism [12, 13]. There is evidence that *Mycobacteria* evade this host response by inhibiting apoptosis, and by stimulating necrosis, which allows the bacteria to disseminate [14, 15]. Furthermore, in an active infection the body’s ability to clear apoptotic cells may be outpaced. The delay in clearance results in the apoptotic cell bodies losing their membrane integrity and becoming secondary necrotic cells [16]. In the case of the apoptosis of an active macrophage, this includes the leaking of lysosomal content. This includes reactive oxygen species (ROS), which leads to systemic inflammation and oxidative stress.

Selenium is an important trace element that has many biological functions, particularly through its incorporation into multiple selenoproteins. There are 25 such proteins in humans [17]. One of these proteins is glutathione peroxidase (GPx), an antioxidant enzyme found in all eukaryotes. This enzyme uses glutathione to reduce hydrogen peroxide, lipid peroxides, and hydroperoxides [18]. Though selenium has been shown to have insulin mimetic properties, elevated selenium has been associated with diabetes [19–21]. This is possibly due to the selenium found in GPx. Elevated GPx, as well as the corresponding elevation in selenium, is associated with type I diabetes [22]. GPx has also recently been implicated in the pathophysiology of type II diabetes. McClung et al. [23] found that overexpression of GPx in mice resulted in the development of hyperinsulinemia, hyperglycemia, and decreased insulin sensitivity, all of which are indicators of type II diabetes. Though the mechanism of this is poorly understood, they proposed that excessive GPx quenched peroxides too quickly, resulting in less ROS-mediated inhibition of protein-tyrosine phosphatases [23]. Inhibition of these phosphatases, which dephosphorylate insulin receptors, increases insulin sensitivity [24]. Xi Yan et al. [25] found that decreasing selenium intake in mice overexpressing GPx decreases the hyperinsulinemia, hyperglycemia, and insulin resistance caused by the elevated GPx expression. Excessive dietary selenium, on the other hand, has been found to upregulate GPx and result in higher insulin resistance [26, 27]. A similar trend was found in humans [28]. Selenoproteins are also upregulated as a result of oxidative stress [29].

The association between infection and GPx activity is poorly understood. The objective of this study was to assess the potential association between MAP infection and the activity of selenium-dependent GPx in bovine and human samples. We hypothesize that the oxidative stress caused by MAP infection will result in elevated GPx activity. The oxidative stress associated with MAP infection may have broad systemic effects and may be clinically relevant to diseases including CD and diabetes.

## Methods

### Bovine samples

Sera samples from healthy and MAP infected cattle were kindly provided by Dr. Michael Collins (University of Wisconsin). Bovine samples were confirmed for MAP infection using the IDEXX *Mycobacterium paratuberculosis (M. pt.)* Antibody Test Kit (IDEXX Laboratories, Westbrook, ME, USA) following manufacturer instructions. A S/P less than or equal to 0.60 was considered negative and a S/P greater than or equal to 0.70 was considered positive. Sera from 21 MAP infected cattle and 21 healthy cattle were then included in this study.

### Human samples

#### Sample processing

Human blood samples were collected in two separate sets where each subject provided three 6.0-ml K_2_-EDTA tubes. All clinical samples were collected following University of Central of Florida-Institutional Review Board approval number IRB00001138. A total of 27 human blood samples were collected from CD patients along with 27 samples of their healthy biological family members (parents or siblings), those samples were collected at the University of Florida (UF). An additional randomized 100 blood samples used in earlier studies were also included. Clinical samples were collected blindly with no prior knowledge of MAP diagnosis or other health conditions. Buffy coat preparations and plasma samples were separated and stored at −20 °C.

#### DNA extraction and nested PCR analysis

DNA extraction for PCR analysis was performed on purified buffy coat samples. Each sample was re-suspended in 100 μL of TE buffer and then incubated at 100 °C for 30 min. The re-suspended solution was then placed in an ice bath for 15 min, after which it was centrifuged for 10 min at 4 °C at 12,000 rpm (18,500*g*). After centrifugation, the supernatant was extracted in 200 μL of phenol/chloroform/isoamyl alcohol (1:1:24 v/v; Acros Organics, Morris Plains, NJ, USA) was added. The solution was mixed and centrifuged for 5 min at 4 °C at 12,000 rpm (18,500*g*). The pellet, containing the nucleic acid, was then washed, dried, and re-suspended in 50 μL of sterile water [3].

Detection of MAP DNA using nested PCR (nPCR) was based on the MAP-specific IS900 derived oligonucleotide primers [3]. As shown in Table 1, P90 and P91 primers were used for the amplification of 398 bp in the first round of amplification and AV1 and AV2 primers, were used to amplify a 298 bp internal sequence. Each primary PCR reaction used 10 µL of DNA template and 40 µL of PCR buffer, which consists of 5 mM MgCl2, 0.2 mM dNTP, 2 µM primers, and 2.5 U Platinum Taq polymerase (Invitrogen, Carlsbad, CA, USA) or 1 U TFL DNA polymerase (Promega, Madison, WI, USA). Each secondary round of PCR used the same ingredients, except different primers were used and 5 µL of the product of the primary round was used instead of the DNA template. Negative controls for the PCR were prepared in which sterile water or TE buffer was added instead of the DNA template (in the primary amplification) or the primary product (in the secondary amplification). These negatives were prepared in parallel with the samples. Positive controls were also prepared using MAP DNA from strain ATCC 43015. The amplification product size was assessed on 2 % agarose gel.

**Table 1:** Primers and amplification conditions used for PCR

Primer	Oligonucleotide sequence (5′–3′)	Gene	Amplification conditions	Product size (bp)	References
P90, P91	GTTCGGGGCCGTCGCTTAGG, GAGGTCGATCGCCCACGTGA	IS900	95 °C for 5 min, then 34 cycles of 95 °C for 1 min, 58 °C for 1.5 min, 72 °C for 1.5 min. Final extension of 10 min at 72 °C	398	Naser et al. [3]
AV1, AV2	ATGTGGTTGCTGTGTTGGATGG, CCGCCGCAATCAACTCCAG	IS900	95 °C for 5 min, then 34 cycles of 95 °C for 1 min, 58 °C for 1.5 min, 72 °C for 1.5 min. Final extension of 10 min at 72 °C	298	Naser et al. [3]

### Selenium-dependent GPx activity measurement

#### Enzyme assay

**Fig. 1:**
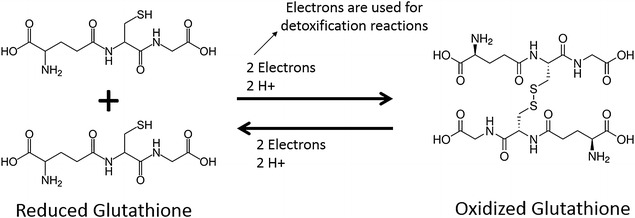
Reduced and oxidized states of glutathione

Glutathione peroxidase works by reducing peroxides by oxidizing glutathione. The glutathione is then restored for further cycles of catalysis (Fig. 1). The rate-limiting step of this reaction is that in which the oxidized glutathione used to reduce the peroxide is restored via the oxidation of NADPH. NADPH absorbs at 340 nm. The selenium-dependent GPx activity was measured by using the Sigma-Aldrich GPx Cellular Activity Assay Kit (Sigma-Aldrich, St. Louis, MO, USA) following manufacturer instructions.

### Statistical analysis

Samples were analyzed for significance using unpaired, two-tailed *t* tests. SigmaPlot software was used. P values of less than 0.05 were considered significant.

## Results

### MAP prevalence in human samples

**Fig. 2:**
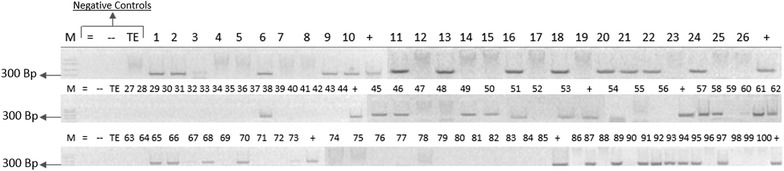
Agarose gels illustrating the presence or absence of MAP-IS900 gene following nPCR. The PCR products following the second round of nPCR were analyzed on 2 % agarose gel. *M* represents molecular weight marker in bp. *=* represents negative control from second round of amplification. − represents negative control from first round of amplification. *TE* represents TE buffer negative control. + represents positive control prepared from MAP DNA strain ATCC 43015. *1–100* represents patient samples

We performed nPCR on DNA extracts isolated from all human blood samples in order to analyze for the presence of MAP-specific IS900 gene according to Naser et al. protocol [3]. The overall prevalence of MAP among 154 human blood samples was 32 %. MAP was positive in the blood of 40 % of CD patients compared to 29.9 % in non-CD patients. Specifically MAP was also positive in 11/27 (40 %) of CD patients and in 2/27 (7 %) in healthy biological family members. Interestingly, 33 % (7 out of 21) of patients with type II diabetes and 44 % (7 out of 16) pre-diabetic patients were also MAP positive. Patients were considered to be pre-diabetic if they had a fasting blood sugar level between 100 and 125 mg/dl, if the two-hour glucose levels was between 140 and 199 mg/dl in an oral glucose tolerance test, or if they had a glycated hemoglobin (A1C) level between 5.7 and 6.4. Figure 2 illustrates the detection of MAP IS900 gene on 2 % agarose gel following nPCR analysis of 100 randomized human blood samples (lanes 1–100).

### Selenium-dependent GPx levels were elevated in MAP infected bovine samples

**Fig. 3:**
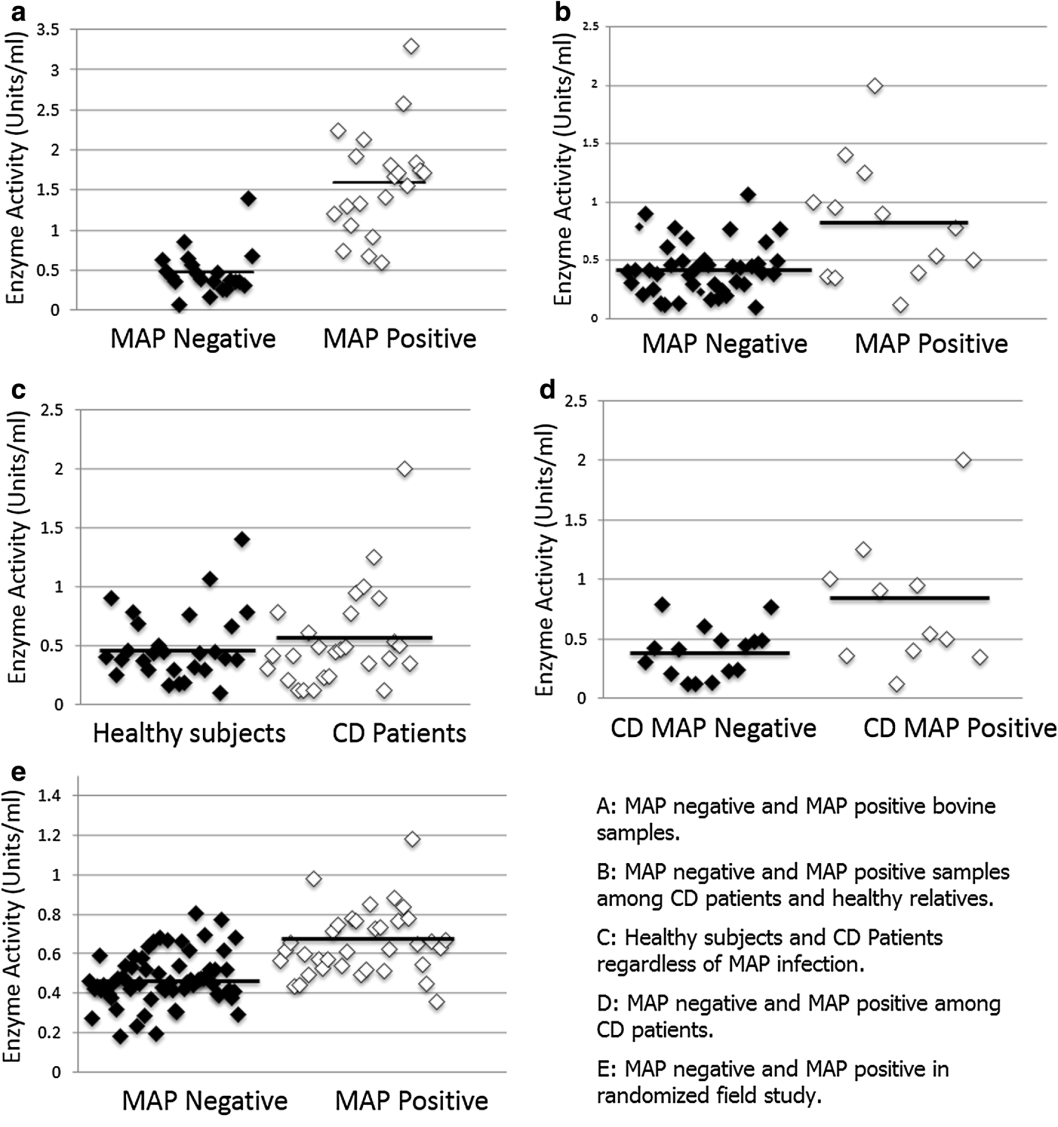
**a** *Scatter plot* of selenium-dependent GPx activity for MAP negative and MAP positive bovine samples. **b** *Scatter plot* of selenium-dependent GPx activity for MAP negative and MAP positive samples among CD patients and healthy relatives. **c** *Scatter plot* of selenium-dependent GPx activity for Healthy and CD individuals. **d** *Scatter plot* of selenium-dependent GPx activity for MAP negative and MAP positive among CD patients. **e** *Scatter plot* of selenium-dependent GPx activity for MAP negative and MAP positive in randomized field study

Bovine sera were confirmed for presence of anti-MAP IgG. Consequently, a total of 21 cattle sera samples from animals diagnosed with Johne’s disease (MAP positive) and 21 sera from healthy cattle (MAP negative) were selected for the study. All 42 sera were analyzed for of GPx activity. The average level of GPx was 0.46907 ± 0.28 units/ml in healthy cattle sera control compared to 1.590 ± 0.65 units/ml in sera from cows infected with MAP, where a unit was defined as one mmol/minute. The MAP positive samples had a significantly higher activity level, with a difference in means of 1.122 (95 % confidence interval 0.810–1.435; P < 0.01) (Table 2). Figure 3a shows a scatter plot of selenium-dependent GPx activity for MAP negative and MAP positive samples.

**Table 2:** GPx enzyme average activity and MAP presence in bovine and human blood samples

Number of samples/total	Source	MAP diagnosis	Average GPx activity (units/ml)	P value
21/42	Bovine	Negative	0.469 ± 0.28	<0.01
21/42	Bovine	Positive	1.590 ± 0.65	
105/154	Human	Negative	0.452 ± 0.176	<0.01
49/154	Human	Positive	0.693 ± 0.30	
16/27	CD patients	Negative	0.389 ± 0.213	<0.05
11/27	CD patients	Positive	0.7593 ± 0.537

### Selenium-dependent GPx activity was elevated in MAP infected humans among Crohn’s patients and their healthy relatives

The average level of GPx activity was 0.80941 ± 0.521 units/ml in the MAP positive samples, while the average enzyme activity in MAP negative samples was found to be 0.42367 ± 0.229 units/ml. This result reveals that MAP infection has a significant influence on GPx activity, with a difference in means of 0.387 (95 % confidence interval 0.182–0.592; P < 0.01) (Fig. 3b).

### The difference between selenium-dependent GPx activity in Crohn’s Disease and in healthy individuals was not significant

In order to confirm that the elevation of GPx activity level was due to MAP infection alone, and not due to CD status, we measured the average of GPx activity in healthy individuals and CD patients separately. The average GPx activity was found to be 0.54 ± 0.414 units/ml and 0.493 ± 0.301 units/ml in CD and healthy patients respectively. While the mean GPx enzymatic activity in CD patients was higher by 0.0469, our results showed that there was no significant difference between both groups (95 % confidence interval −0.245 to 0.151; P = 0.636) (Fig. 3c). The gender ratio and age distribution between the two groups was comparable between the two groups (Table 3).

**Table 3:** Demographics of Crohn’s patients and healthy relatives

Group	Age range	Average age	Gender ratio (M/F)
Relatives	12–65	45	9/18
Crohn’s	16–56	32	8/19

### Selenium-dependent GPx activity was elevated in MAP infected Crohn’s patients

As mentioned earlier, out of 27 CD patients, a total of 11 were tested as MAP positive, while 16 were MAP negative. The average GPx activity in CD patients who had the MAP infection was 0.7593 ± 0.537 units/ml, while the GPx activity was found to be 0.389 ± 0.213 units/ml in CD patients without MAP infection. The difference in means was 0.37 (95 % confidence interval 0.07–0.675; P = 0.019). (P = 0.019) (Fig. 3d). Furthermore only 2 of the 27 healthy relatives used as controls, or 7.4 %, were infected with MAP.

### Selenium-dependent GPx activity was elevated among MAP infected humans in randomized field study

**Fig. 4:**
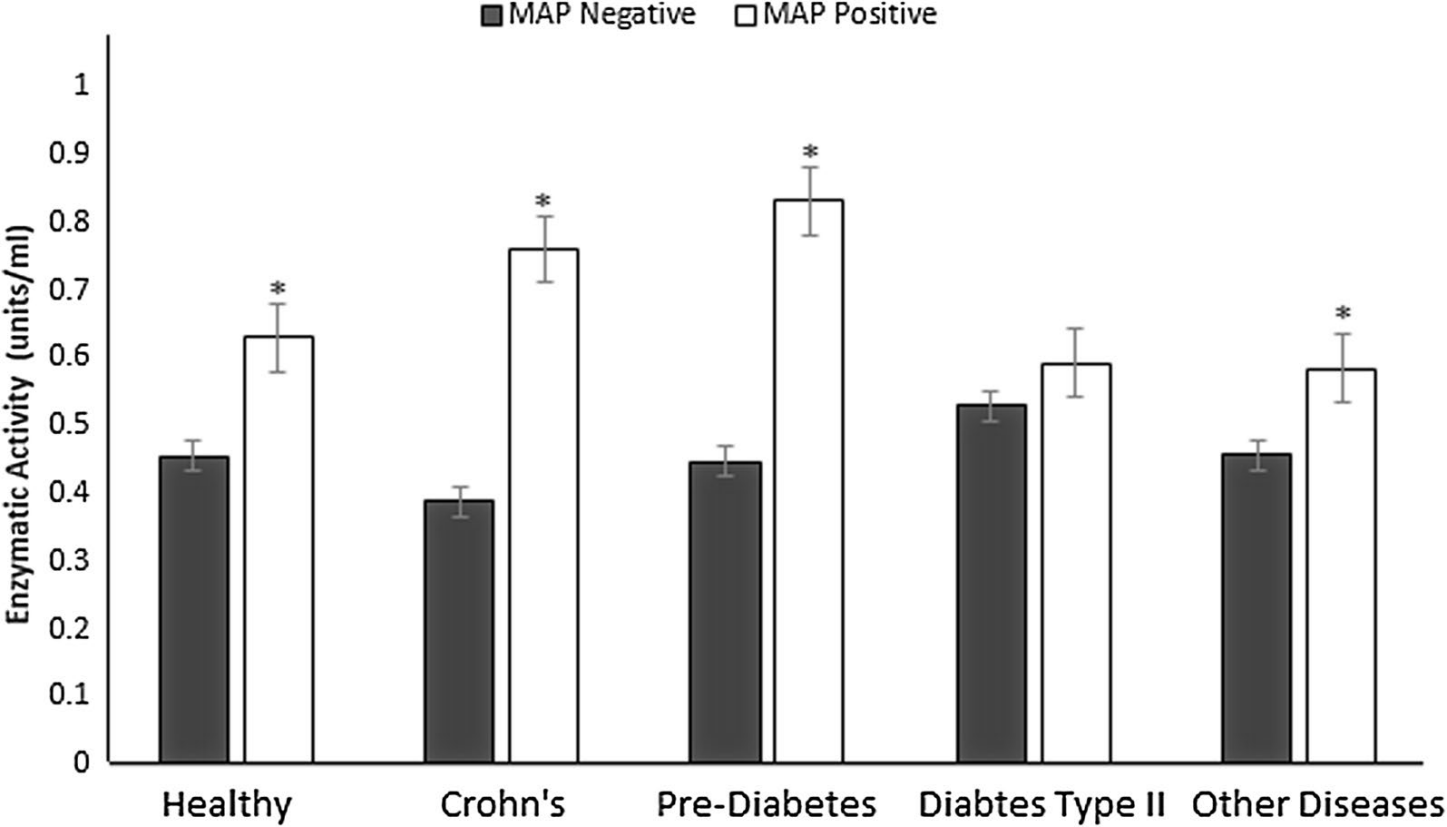
Average GPx activity levels in plasma samples from blood samples identified as as MAP negative and positive individuals according to according to disease status

Among randomized blood samples from 100 subjects, 36 were determined to be MAP positive as shown in Fig. 2. The average of GPx activity level in 36 MAP positive clinical samples was 0.6510 ± 00.1665 units/ml compared 0.4702 ± 0.1299 in 64 MAP negative clinical samples (P < 0.01) (Table 2). The GPx activity in each clinical sample is illustrated in Fig. 3e. We further examined the difference in GPx activity according to disease diagnosis, but there was no significant difference in MAP negative clinical samples between healthy controls and subjects with diseases. Disease states, including type 2 diabetes and pre-diabetes, were not found to have a significant impact on GPx activity. It is notable, however, that in all disease states MAP positive individuals still have higher enzymatic activity than MAP negative individuals (Fig. 4).

## Discussion

Oxidative stress and the resulting GPx up-regulation may have significant implications on disease pathophysiology. In particular, long-term up-regulation of GPx may cause insulin resistance and disruptions in insulin signaling [23]. Furthermore, oxidative damage has recently been an area of focus for research into inflammatory conditions such as CD and inflammatory bowel disease (IBD). Despite this, the relationship between MAP infection and oxidative stress has not been clearly established. The purpose of our study was therefore to elucidate the relationship between MAP infection and GPx activity in plasma. This makes it possible to find the differences in oxidative stress level in CD patients with the presence and absence of MAP infection, which may have implications on the treatment of these patients. We acquired bovine and human samples, tested them for MAP infection, and measured their GPx activity.

We found that the enzymatic activity of GPx was significantly higher in cows as well as among two separate groups of human subjects that are infected with MAP, where significance was defined as P < 0.05. This matched the expected trend. We chose to keep the data for the two cohorts of human patients separate, as the samples for these two groups were collected separately and for different reasons. The CD patients and their healthy relatives were used primarily to determine if the observed trend was caused by CD status, or if it was caused by MAP infection. We propose to exclude CD as a potential cause of the observed trend due to the previously established relationship between MAP and CD [1, 7, 30–33]. The biological relationships present in this group was ideal for determining the effects of CD, as any observable difference in GPx activity could be attributed to the disease status, rather than hereditary environmental factors. However, in determining the effect of MAP on GPx activity, we determined that a randomized study would be preferable, and would better reflect the prevalence of MAP in subjects at large. It is worth noting, however, that when all human clinical samples were combined, MAP positive samples still had significantly elevated GPx activity when compared to MAP negative samples (P < 0.01).

We found that there was there no significant difference in GPx activity in MAP negative samples from CD and non-CD samples. However, GPx activity was elevated in all clinical samples which were positive for MAP regardless of the source of samples. While it was surprising that an inflammatory condition like CD, as well as other disease states, has no significant effect on GPx, this makes a stronger case that MAP infection may be playing a role in the elevation in enzymatic activity. We further excluded CD status as a potential confounding factor in the human samples by comparing CD patients infected with MAP with those not infected with MAP. We found that there was still a significant correlation between MAP infection and GPx even when CD status was controlled for (P < 0.05).

The prevalence of MAP in CD patients was 40 % compared to 7.4 % MAP in healthy relatives (P < 0.01), indicating that the CD patients were more susceptible to MAP infection. This is consistent with published reports [7, 30–32, 34–36]. This study is the first to investigate the association of MAP and CD did using healthy relatives as controls. Our findings strengthen the growing body of literature supporting the correlation between MAP and CD. Of course the role of MAP in CD etiology remains debated but genetic susceptibility especially single nucleotide polymorphism in key genes in patients with CD may promote MAP infection [7, 34–36].

The randomized component of this study revealed a 36 % MAP prevalence. Since these patients were randomly selected, this is likely to reflect the true prevalence of MAP infection in the community. The differences in MAP prevalence between the healthy relatives of Crohn’s patients and the community at large may be due to complex genetic and environmental factors yet to be elucidated, and which warrant further study. It is worth noting that MAP has been associated with several autoimmune diseases including CD, T1D, Blau syndrome, Hashimoto’s thyroiditis, and multiple sclerosis [37–41]. In fact, Sechi and Dow [39] have discussed in a recent review article that many subjects among us might be positive for MAP because of suffering from other diseases where MAP might be a culprit. The cross reactivity between MAP antigens and host proteins in patients with these diseases could be the base for molecular mimicry where exposure to MAP could lead to reprograming of the immune system and ultimately leading to production of autoantibodies and tissue damage. It is a fact that MAP is wide-spread in the environment and now is part of the food chain and genetic predisposition in key genes invites susceptibility to MAP infection. Interestingly, Sechi’s group has recently reported that epitope homology between human interferon regulatory factor 5 of Epstein-Barr virus and *M. avium* subsp. paratuberculosis induces a specific humoral and cellular immune response in multiple sclerosis patients [44]. This report expands on the molecular mimicry between pathogens versus host to branch out molecular mimicry to be between pathogens. Consequently, remote MAP infection in humans may be possible. So the lack of clinical data on controls especially with regard to diagnosis for other diseases may play a factor in the prevalence of MAP in subjects included in this study.

The pathogenesis mechanism of CD involves interaction between environmental agents, genetic and immunologic abnormalities [42]. Recently, the role of reactive oxygen species has been an area of interest to study IBD pathophysiology [43]. Granulocyte accumulation is increased in the gut mucosa where inflammation is active in IBD patients and those cells secrete different inflammatory mediators [42]. It has been shown that mucosal inflammation impairs antioxidant defense and the tissues become more liable to oxidative damage [44]. Increasing GPx antioxidant activity is a result of elevation in free radical levels. It is unknown if granulocyte accumulation is increased in CD patients who are infected with MAP in comparison to CD patients who are not. This will lead to different phenotypes of CD patients who are MAP positive or negative with differences in oxidative stress and free radical levels in according to the disease state which will be reflected in the clinical status of those patients. In T1D, a significant homology was reported between human glutamic Acid decarboxylase 65 (GAD65) and MAP HSP65 [37, 38]. This cross reactivity supports molecular mimicry between host and microbe interaction [37]. Future studies are also needed to evaluate the enhanced disease state that may be present in MAP infected Crohn’s patients and those considered controls.

We also considered smoking, gender, and age as potential confounding factors. There was no significant difference in GPx activity between males and females or smoking habits. The GPx enzymatic activity of patients above 40 was overall lower, but the difference was not significant. Overall there was no significant correlation between age and GPx activity (r = 0.0648, P = 0.74). None of these factors proved to have an effect on our data.

While significant in both human and bovine samples, the difference in average GPx activity between MAP infected and non-infected samples was much more extreme in the bovine samples. This is possibly because bovine are capable of a more robust response, or because they have a larger bacterial load, than humans. There are also less confounding factors in bovine samples, as cattle used as livestock are fed similar diets and live in similar conditions, as opposed to humans in which these factors vary widely. As such, cows may be a purer model for study, and the results from the bovine samples may be more representative.

The unique progression of MAP infection causes systemic inflammation and oxidative stress. It is possible that GPx production is up-regulated in order to compensate for this. Though on the short term this may offset the negative effects of the infection, on the long term, particularly in chronic infections, it may cause its own problems. While the long-term oxidative stress caused by MAP infection may not be fatal, it may have a wide range of deleterious systemic effects. It is possible that other species of *Mycobacteria* have similar effects. Future studies will further elucidate the relationship between MAP infection, GPx up-regulation, glucose homeostasis, as well as the clinical status of CD patients.

## Conclusion

The GPx enzymatic activity of selenium dependent GPx was significantly higher in both bovine and human serum samples infected with MAP. The consistent correlation between MAP infection and GPx activity potentially may be used to predict MAP infection status. We posit that the presence of this bacterium causes systemic inflammation and oxidative stress, which on the long-term may cause disruptions in insulin signaling and have a deleterious effect on insulin sensitivity. Via this process MAP infection could be involved in the pathophysiology of insulin resistance and in the elevation of oxidative stress level in CD patients who are infected with MAP.
